# Differential Cytotoxicity but Augmented IFN-γ Secretion by NK Cells after Interaction with Monocytes from Humans, and Those from Wild Type and Myeloid-Specific COX-2 Knockout Mice

**DOI:** 10.3389/fimmu.2015.00259

**Published:** 2015-06-09

**Authors:** Han-Ching Tseng, Aida Arasteh, Kawaljit Kaur, Anna Kozlowska, Paytsar Topchyan, Anahid Jewett

**Affiliations:** ^1^Division of Oral Biology and Oral Medicine, The Jane and Jerry Weintraub Center for Reconstructive Biotechnology, UCLA School of Dentistry and Medicine, Los Angeles, CA, USA; ^2^Department of Tumor Immunology, Poznan University of Medical Sciences, Poznan, Poland; ^3^The Jonsson Comprehensive Cancer Center, UCLA School of Dentistry and Medicine, Los Angeles, CA, USA

**Keywords:** COX-2, NK, IFN-γ, cytotoxicity, LPS

## Abstract

The list of genes, which augment NK cell function when knocked out in neighboring cells is increasing, and may point to the fundamental function of NK cells targeting cells with diminished capability to differentiate optimally since NK cells are able to target less differentiated cells, and aid in their differentiation. In this paper, we aimed at understanding the effect of monocytes from targeted knockout of COX-2 in myeloid cells (*Cox-2^flox/flox^;LysM^Cre/^*^+^) and from control littermates (*Cox-2^flox/flox^;LysM*^+^*^/^*^+^) on *ex vivo* function of NK cells. Furthermore, we compared the effect of monocytes treated with and without lipopolysaccharide (LPS) on NK cells from mice and humans. NK cells purified from *Cox-2^flox/flox^;LysM^Cre/^*^+^ mice had heightened cytotoxic activity when compared to those obtained from control littermates. In addition, NK cells cultured with autologous *Cox-2^flox/flox^;LysM^Cre/^*^+^ monocytes and DCs, mouse embryonic fibroblasts from global knockout COX-2, but not with knockout of COX-2 in T cells, had increased cytotoxic function as well as augmented IFN-γ secretion when compared to NK cells from control littermates cultured with monocytes. LPS inhibited NK cell cytotoxicity while increasing IFN-γ secretion when cultured in the presence of monocytes from either *Cox-2^flox/flox^;LysM^Cre/^*^+^ or control littermates. In contrast to mice, NK cells from humans when cultured with monocytes lost cytotoxic function and gained ability to secrete large amounts of IFN-γ, a process, which we had previously coined as “split anergy.” Similar to mice, LPS potentiated the loss of human NK cell cytotoxicity while increasing IFN-γ secretion in the presence of monocytes. Greater loss of cytotoxicity and larger secretion of IFN-γ in NK cells induced by gene knockout cells may be important for the greater need of these cells for differentiation.

## Introduction

Knockout mouse models have provided a powerful tool for the identification and elucidation of mechanisms underlying different physiological and pathological processes in mice. However, a closer look at several of gene knockout mouse models (Table 1) revealed the pro-inflammatory nature of the immune responses in these animals. Ironically, increased inflammatory responses can also be detected in mice with knockouts of genes that affect the core function of cells, which mediate inflammation (1). In addition, we have previously reported knockout of key genes in healthy, as well as transformed human oral tumors increased the functional activation of Natural Killer (NK) cells (2–4).

**Table 1:** **Heightened immune cell function and increased inflammation in a variety of gene knockout mice**.

Gene	Reference
NF-κB	(2–4)
STAT3	(5–7)
CD133	(8)
NEMO	(9–11)
TNF-α	(12)
DAP10/DAP12	(1)
Clc-5	(13)
MCP-1	(14)
Transglutaminase 3	(15)
Presenilins 1 and 2	(16)
Annexin-1	(17)
A20 (TNFAIP3)	(18)
Galectin-3	(19)
PGC-1α	(20)
LDLR	(21)
Abca1	(22)
Cprc5a	(23)
BCMO1	(24)
PAP/HIP	(25)

The prostanoids, which include prostaglandins, prostacyclins, and thromboxanes, modulate several important physiological and pathophysiological processes such as gastric mucosal integrity (26, 27), vasodilation (28, 29), allergic response (30, 31), platelet adhesion and aggregation (32, 33), wound healing (34–36), and water balance (37). Cyclooxygenase (COX), the enzyme required to generate prostanoids, exists in two isoforms, COX-1 and COX-2. COX-1 is constitutively expressed in most tissues at a consistent level and dysregulation results in gastric damage, bleeding, and ulceration (38, 39). Under normal conditions, the COX-2 activity is low and upon induction by growth factors and pro-inflammatory cytokines, the protein expression can be up- or down-regulated within hours (40, 41). The involvement of COX-2 in tumor development and progression has also been demonstrated in numerous cancer types (42–44).

NK cells are lymphocytes that arise from the bone marrow and are capable of mediating direct natural cytotoxicity and antibody-dependent cellular cytotoxicity. NK cells are identified by the expression of CD16 and CD56 in humans and DX5 or NK1.1 in mice and lack surface expression of CD3. NK cells mediate cytotoxicity against a variety of malignant tumors, virally infected cells, as well as healthy untransformed, undifferentiated cells (45, 46). Many significant differences, both in function and phenotype of NK cells from mice and humans, have been identified in previous reports.

Our laboratory has coined the term “split anergy” as a condition in which NK cells lose cytotoxicity and gain the ability to secrete cytokines (47–53). Split anergy is initiated by the receptor triggering of CD16, NKp46, toll-like receptors (TLRs), and interaction with both healthy and transformed stem-like cells, as well as with other immune effectors such as monocytes and with fibroblasts in humans (54–57), and in this report we present similar effect in mice. It has been reported that human NK cells can become activated by the membrane component of gram-negative bacteria lipopolysaccharide (LPS) which serves as a TLRs-4 ligand. NK cells can proliferate either as a result of indirect interaction with LPS-treated macrophages and dendritic cells (DCs) or directly via TLR triggering on NK cells (58, 59). Since NK cells are important effectors of selection and differentiation of stem cells, the induction of split anergy in NK cells is a physiologically important step in converting the phenotype of NK cells from cytotoxic to those of cytokine producing cells, allowing differentiation of the stem cells. This enables the NK cells not only to remove defective stem cells but also to limit the size and proliferation of stem cells, in addition to the promotion of differentiation of selected stem cells (54–56, 60, 61).

Our previous data and those from others collectively suggest that the inhibition of genes involved in differentiation of tumors result in an increase in cytotoxic function of NK cells (2–7). The list of cellular genes, which augment cytotoxicity in NK cells when deleted or decreased in tumors is increasing, and may point to a fundamental function of NK cells targeting cells, which lose ability to differentiate optimally since NK cells are known to target poorly differentiated/stem-like cells. Therefore, in this paper we aimed at understanding the effect of the deletion of COX-2, another important differentiation gene in monocytes, on the function of NK cells from mice carrying the targeted knock down of COX-2 in myeloid cells. In addition, the function of NK cells after interaction with monocytes from mice and humans were compared.

## Materials and Methods

### Mice

Myeloid cell-specific COX-2 targeted knockout mice (*Cox-2^flox/flox^;LysM^Cre/^*^+^) and their control wild type (WT) littermates (*Cox-2^flox/flox^;LysM*^+^*^/^*^+^), as well as global COX-2 knockout (COX-2^−/−^) and their control wildtype littermates were generated and bred at UCLA in Dr. Harvey Herschman’s laboratory and used for this study (62). C57BL/6 mice were purchased from Jackson Laboratory (Bar Harbor, ME, USA).

### Cell lines, reagents, and antibodies

RPMI 1640 supplemented with 10% Fetal Bovine Serum (FBS) was used for the cultures of human NK cells, monocytes, and mouse NK cells, T cells, monocytes and DCs. RPMI 1640 supplemented with 10% FBS was also used to culture mouse T cell lymphoma (YAC-1). ST63 cells were cultured in RPMI 1640 supplemented with 10% FBS. COX-2 wild type and COX-2 knockout Mouse Embryonic Fibroblasts (MEFs) were cultured in DMEM supplemented with 10% FBS (62). Oral Squamous Cancer Stem Cells (OSCSCs) were isolated from the tongue tumors of the patients at UCLA and cultured in RPMI 1640 supplemented with 10% FBS (Gemini Bio-Products, CA), 1.4% antibiotic antimycotic, 1% sodium pyruvate, 1.4% non-essential amino acids, 1% l-glutamine, 0.2% gentamicin (Gemini Bio-Products, CA, USA) and 0.15% sodium bicarbonate (Fisher Scientific, PA, USA). IFN-γ was purchased from Biolegend (San Diego, CA, USA) and TNF-α was purchased from PeproTech (Rocky Hill, NJ, USA). LPS was purchased from Sigma-Aldrich (St. Louis, MO). IL-4 and GM-CSF were purchased from Biolegend (San Diego, CA, USA) and used to differentiate purified monocytes into DCs. Recombinant IL-2 was obtained from NIH-BRB. Antibodies to CD16, B7H1, CD45, CD54, DX5, Ly49A, Ly49D, Rae-1γ, NKG2D, and F4/80 were purchased from Biolegend (San Diego, CA, USA). Antibody to MHC class-I was purchased from eBioscience (San Diego, CA, USA). Flow cytometry analysis was performed using Beckman Coulter Epics XL cytometer (Brea, CA, USA) and results were analyzed in FlowJo vX software (Tree Star, Ashland, OR, USA). The mouse and human NK cells, T cells, and monocyte purification kits were obtained from Stem Cell Technologies (Vancouver, BC, Canada).

### Bacterial preparation

AJ2 is a combination of eight gram-positive bacterial strains (*Streptococcus thermophilus, Bifidobacterium longum, Bifidobacterium breve, Bifidobacterium infantis, Lactobacillus acidophilus, Lactobacillus Plantarum, Lactobacillus casei, and Lactobacillus bulgaricus)* each selected and combined for the optimal capability to induce differentiation of stem cells (60) (manuscript submitted). AJ2 was re-suspended in RPMI supplemented with 10% FBS (Gemini Bio-Products, CA) at a final concentration of 10 mg/mL. The bacteria were then sonicated using ultra-sonicator for 15 s while on ice. Afterward, the sonicated bacteria were incubated for 30 s on ice. The sonication process was repeated 20 times to achieve complete sonication. Lastly, the sonicated samples (sAJ2) were aliquoted and stored in −80° freezer until use.

### Purification of human NK cells and monocytes

Written informed consents approved by UCLA Institutional Review Board (IRB) were obtained from the blood donors and all the procedures were approved by the UCLA-IRB. NK cells from healthy donors were isolated as described before (51). Briefly, peripheral blood lymphocytes were obtained after Ficoll-hypaque centrifugation and purified NK cells were negatively selected by using an NK cell isolation kit (Stem Cell Technologies, Vancouver, BC, Canada). The purity of NK cell population was found to be >90% based on flow cytometric analysis of anti-CD16 antibody stained cells. The levels of contaminating CD3^+^ T cells remained low, at 2.4 ± 1%, similar to that obtained by the non-specific staining using isotype control antibody throughout the experimental procedures. The adherent subpopulation of PBMCs was detached from the tissue culture plates and monocytes were purified using isolation kit obtained from Stem Cell Technologies (Vancouver, BC, Canada). Greater than 95% purity was achieved based on flow cytometric analysis of CD14 antibody stained monocytes.

### Mouse NK cells, T cells, monocytes and dendritic cell cultures

All animal work performed was based on the guidelines established and approved by UCLA Office of Animal Research Oversight. Single cell preparations of mouse splenocytes were used to negatively select mouse NK cells using mouse NK isolation kit purchased from Stem Cell Technologies (Vancouver, Canada). The purity of mouse NK cells were >90% based on staining with PE-conjugated DX5 antibody (Figure S1 in Supplementary Material). NK cells were treated with IL-2 (1 × 10^4^ U/million NK cells) for 7 days before the cells were used for experiments. T cells were purified using mouse T cell isolation kit purchased from Stem Cell Technologies (Vancouver, BC, Canada). Bone marrow cells were isolated by flushing femurs with PBS supplemented with 2% heat-inactivated FBS. Murine monocytes were then purified from bone marrow cells using monocyte isolation kit obtained from Stem Cell Technologies (Vancouver, BC, Canada). The purity of monocytes was between 86 and 96% based on staining with PE-conjugated anti-CD14 antibody. To differentiate mouse DCs from purified monocytes, IL-4 (20 ng/mL) and GM-CSF (20 ng/mL) were added to monocytes for 7 days.

### ELISA and multiplex assays

Single ELISAs were performed as described previously (51). Fluorokine MAP cytokine multiplex kits were purchased from R&D Systems (Minneapolis, MN, USA) and the procedures were conducted as suggested by the manufacturer. To analyze and obtain the cytokine and chemokine concentration, a standard curve was generated by either two- or threefold dilution of recombinant cytokines provided by the manufacturer. Analysis was performed using the Star Station software. Samples were analyzed using Beckman Coulter EPICS XL cytometer and subsequently analyzed in FlowJo software (Tree Star, Ashland, OR, USA).

### ^51^Cr release cytotoxicity assay

The ^51^Cr release assay was performed as described previously (3). Briefly, different numbers of purified NK cells were incubated with ^51^Cr–labeled target cells. After a 4 h incubation period, the supernatants were harvested from each sample and counted for released radioactivity using the gamma counter. The percentage specific cytotoxicity was calculated as follows:

$$\%\text{Cytotoxicity}=\frac{\text{Experimental cpm}-\text{spontaneous cpm}}{\text{Total cpm}-\text{spontaneous cpm}}$$

LU 30/10^6^ is calculated by using the inverse of the number of effector cells needed to lyse 30% of tumor target cells × 100.

### Statistical analysis

An unpaired, two-tailed student *t*- test was performed for the statistical analysis. One-way ANOVA with a Bonferroni post-test was used to compare the different groups.

## Results

### NK cells derived from *Cox-2^flox/flox^;LysM^Cre/+^* mice mediated higher cytotoxicity

**Figure 1:**
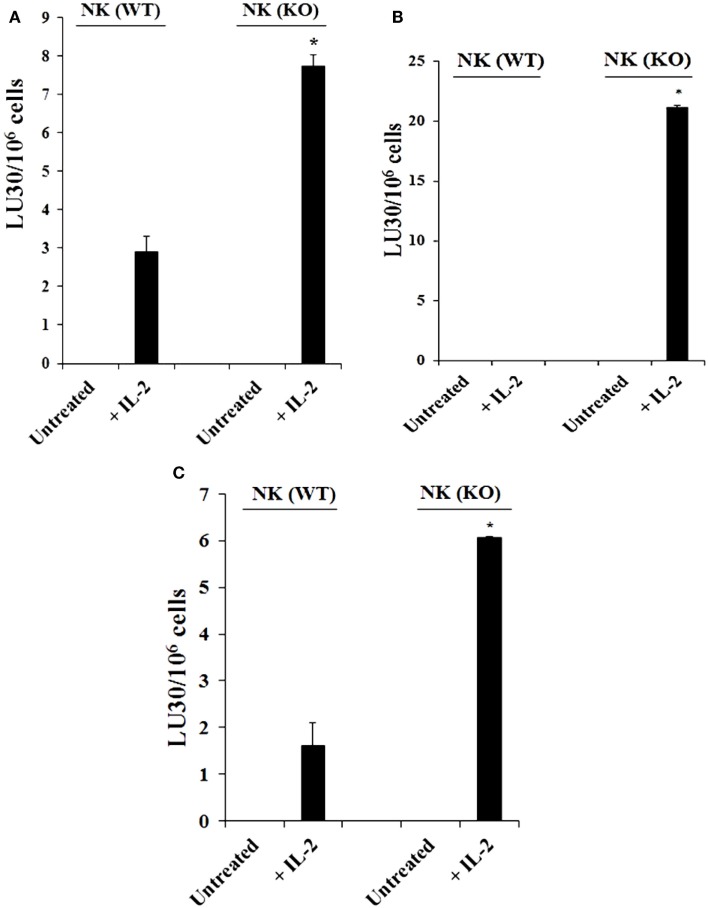
**Increased cytotoxicity by NK cells derived from *Cox-2^flox/flox^;LysM^Cre/^*^+^mice compared to those obtained from control WT littermates**. Purified NK cells obtained from either control (WT) or *Cox-2^flox/flox^;LysM^Cre/^*^+^ (KO) mice were left untreated or treated with IL-2 (1 × 10^4^ U/million) for 7 days before they were used against YAC-1 cells **(A)**, Mouse Embryonic Fibroblasts **(B)**, and ST63 **(C)** in a standard 4 h ^51^Chromium release assay. The lytic units 30/10^6^ cells were determined using inverse number of NK cells required to lyse 30% of the target cells × 100. *P < 0.05 was obtained for the difference between control WT *Cox-2^flox/flox;^LysM^Cre/^*^+^ NK cell cytotoxicity against YAC-1 cells, MEFs, or ST63. One of several representative experiments is shown in this figure.

Purified NK cells obtained from spleens of control WT littermates (*Cox-2^flox/flox^;LysM*^+^*^/^*^+^) and those with targeted knockout of COX-2 gene in myeloid cells (*Cox-2^flox/flox^;LysM^Cre/^*^+^) were left untreated or treated with IL-2 for 7 days before they were used in a standard ^51^Cr release assay against YAC-1 cells (Figure 1A; Figure S2A in Supplementary Material), Mouse Embryonic Fibroblasts (MEFs) (Figure 1B), and ST63 cells (Figure 1C; Figure S2B in Supplementary Material). As shown in Figure 1, purified IL-2-treated NK cells from *Cox-2^flox/flox^;LysM^Cre/^*^+^ mice lysed YAC-1 (*P* < 0.05), MEFs (*P* < 0.05), and ST63 cells (*P* < 0.05) significantly more than IL-2-treated NK cells from control WT littermates which had no/low cytotoxicity. Untreated NK cells did not mediate any cytotoxicity (Figure 1; Figure S1 in Supplementary Material).

### NK cells obtained from *Cox-2^flox/flox^;LysM^Cre/+^* mice cultured with autologous monocytes mediated significantly higher levels of cytotoxicity than those from control littermates cultured with and without monocytes

**Figure 2:**
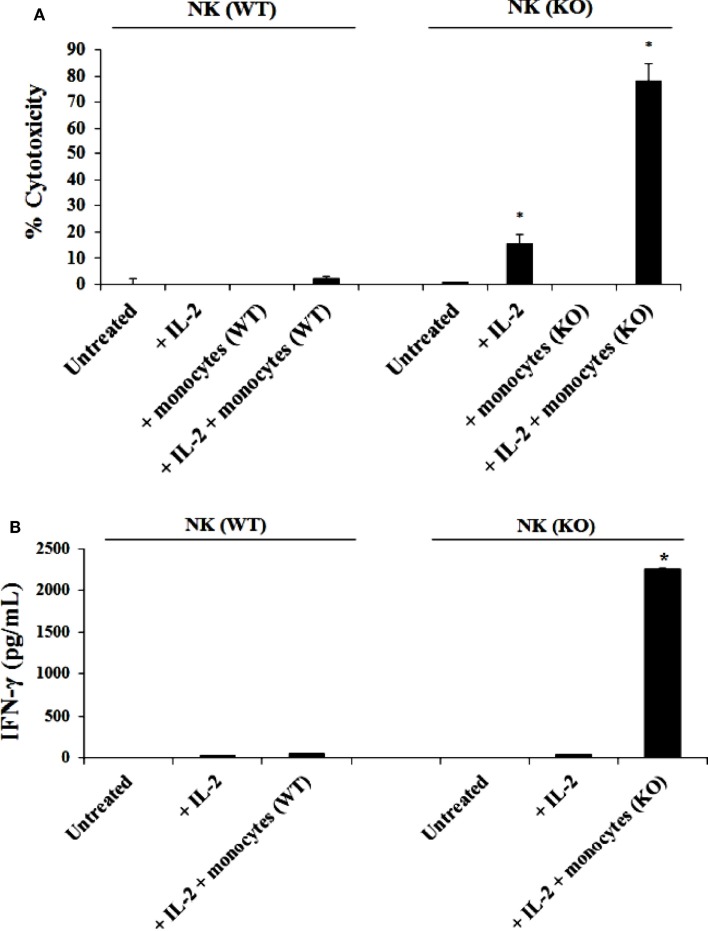
**IL-2 activated NK cells from *Cox-2^flox/flox^;LysM^Cre/^*^+^ mice cultured with autologous monocytes lysed YAC-1 cells and secreted high levels of IFN-γ as compared to NK cells from control littermates in the presence and absence of autologous monocytes**. NK cells obtained from control mice or *Cox-2^flox/flox;^LysM^Cre/^*^+^mice were left untreated or treated with IL-2 (1 × 10^4^ U/million) in the presence or absence of autologous monocytes for 7 days. Afterward, the cytotoxic function of NK cells against YAC-1 cells was determined using a standard 4 h ^51^Chromium release assay. *P < 0.05 was obtained for the difference in cytotoxicity against YAC-1 tumors mediated by IL-2-treated NK cells cultured with or without monocytes between control and *Cox-2^flox/flox^;LysM^Cre/^*^+^ mice **(A)**. NK cells were treated as described in **(A)** Afterward, the supernatants were removed from the co-cultures and the levels of IFN-γ secretion were determined using specific ELISAs **(B)**. *P < 0.05 was obtained for the difference in IFN-γ secretion from IL-2-treated NK cells between control and *Cox-2^flox/flox^;LysM^Cre/^*^+^ mice cultured with monocytes. One of several representative experiments is shown in this figure.

Purified NK cells from control WT littermates and *Cox-2^flox/flox^;LysM^Cre/^*^+^ mice were cultured with or without purified autologous bone marrow derived monocytes for 7 days before the cells were used in a standard 4 h ^51^Cr release assay against YAC-1 tumors (Figure 2A). As shown in Figure 2A, IL-2-treated NK cells from control WT mice cultured with autologous monocytes mediated slightly higher cytotoxicity compared to the NK cells cultured without monocytes. IL-2-treated NK cells purified from *Cox-2^flox/flox^;LysM^Cre/^*^+^ mice cultured with autologous monocytes lysed YAC-1 cells significantly more compared to NK cells cultured without autologous monocytes, and those purified from control WT animals cultured with autologous monocytes (*P* < 0.05). IL-2-treated NK cells cultured with autologous monocytes from *Cox-2^flox/flox^;LysM^Cre/^*^+^ mice also exhibited higher cytotoxicity against transformed mouse oral keratinocytes and MC38 cells (data not shown) as compared to NK cells from control WT littermates cultured with autologous monocytes.

### NK cells purified from *Cox-2^flox/flox^;LysM^Cre/+^* mice cultured with autologous monocytes produced significantly higher IFN-γ than those from control WT littermates cultured with and without autologous monocytes

Purified NK cells obtained from *Cox-2^flox/flox^;LysM^Cre/^*^+^ mice and control WT littermates were cultured with or without purified autologous monocytes for 7 days, after which the supernatants were collected and the levels of IFN-γ produced by NK cells were measured with specific ELISA. Untreated NK cells did not secrete IFN-γ (Figure 2B). IL-2 treated NK cells from both control WT and *Cox-2^flox/flox^;LysM^Cre/^*^+^ mice produced much lower levels of IFN-γ in the absence of autologous monocytes (Figure 2B). Significantly higher levels of IFN-γ were secreted by NK cells from *Cox-2^flox/flox^;LysM^Cre/^*^+^ mice when cultured with autologous monocytes, whereas much lower amounts of IFN-γ could be seen in supernatants from NK cells from control WT littermates cultured with autologous monocytes (*P* < 0.05) (Figure 2B).

### Step wise increase in cytotoxicity and IFN-γ secretion when NK cells from control WT mice or *Cox-2^flox/flox^;LysM^Cre/+^* mice were cultured with wild type or COX-2^−/−^ monocytes, respectively

**Figure 3:**
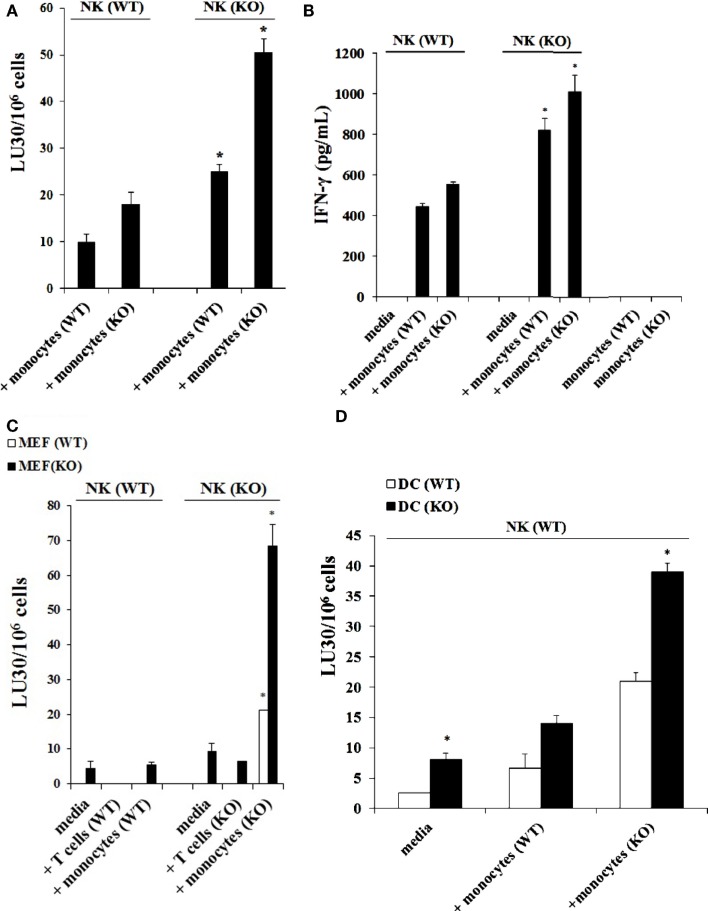
**Monocytes, and not T cells, from *Cox-2^flox/flox^;LysM^Cre/^*^+^ mice enhanced the cytotoxic function of autologous NK cells and induced high levels of IFN-γ secretion**. Wild type or *Cox-2^flox/flox^; LysM^Cre/^*^+^ derived NK cells were activated with IL-2 (1 × 10^4^ U/million) and cultured with either wild type or *Cox-2^flox/flox^;LysM^Cre/^*^+^ monocytes for 7 days. Afterward, the cytotoxic function of NK cells against YAC-1 was determined using a standard 4 h ^51^Chromium release assay. The lytic units 30/10^6^ cells were determined using inverse number of NK cells required to lyse 30% of the target cells × 100. *P < 0.05 is for the difference in cytotoxicity against YAC-1 tumors between IL-2-treated NK cells from control and *Cox-2^flox/flox^;LysM^Cre/^*^+^ mice cultured with monocytes **(A)**. NK cells were prepared as described in (A) and then supernatants from NK cell cultures were harvested after co-incubation with monocytes for 7 days. Monocytes from wild type and *Cox-2^flox/flox^;LysM^Cre/^*^+^ mice were used as control. The levels of IFN-γ secretion were determined using specific ELISAs. *P < 0.05 is for the difference in IFN-γ secretion between IL-2-treated NK cells from control and *Cox-2^flox/flox;^LysM^Cre/^*^+^ mice cultured with monocytes **(B)**. NK cells were treated with IL-2 (1 × 10^4^ U/million) and cultured with either T cells from global COX-2 knockout mice or monocytes from wild type or *Cox-2^flox/flox^;LysM^Cre/^*^+^ mice for 7 days. Afterward, NK cells were used as effectors against wild type MEFs or MEFs with specific COX-2 deletion. The cytotoxic function of NK cells against MEFs was determined using a standard 4 h ^51^Cr release assay. The lytic units 30/10^6^ cells were determined using inverse number of NK cells required to lyse 30% of the target cells ×100. *P < 0.05 is for the difference in cytotoxicity between IL-2-treated NK cells from control and *Cox-2^flox/flox^;LysM^Cre/^*^+^ mice cultured with monocytes or T cells **(C)**. IL-2-treated (1 × 10^4^ U/million) NK cells obtained from wild type mice were cultured with monocytes from wild type mice or *Cox-2^flox/flox^;LysM^Cre/^*^+^ mice for 7 days before the cells were used as effector cells in a standard 4 h ^51^Chromium release assay. Monocyte-derived DCs from wild type or *Cox-2^flox/flox^;LysM^Cre/^*^+^ mice were prepared as described in Section “Materials and Methods” and used as target cells. The lytic units 30/10^6^ cells were determined using inverse number of NK cells required to lyse 30% of the target cells × 100. *P < 0.05 was obtained for the difference in IL-2-treated NK cell-mediated lysis between DCs from control mice and from those of *Cox-2^flox/flox^;LysM^Cre/^*^+^ mice **(D)**. One of several representative experiments is shown in this figure.

NK cells purified from either control WT littermates or *Cox-2^flox/flox^;LysM^Cre/^*^+^ mice and treated with IL-2 were co-cultured with either wild type monocytes or monocytes from *Cox-2^flox/flox^;LysM^Cre/^*^+^ mice. The cytotoxic function of NK cells obtained from wild type mice against YAC-1 tumors remained at the lowest when cultured with wild type monocytes whereas an increase in the levels of cytotoxicity could be observed when they were cultured with monocytes from *Cox-2^flox/flox^;LysM^Cre/^*^+^ mice (Figure 3A). NK cells from *Cox-2^flox/flox^;LysM^Cre/^*^+^ mice exhibited higher cytotoxicity either cultured with wild type monocytes (*P* < 0.05) or COX-2 knockout monocytes (*P* < 0.05) as compared to NK cells from wild type mice, although the levels were much higher when cultured with COX-2 knockout monocytes (Figure 3A). Therefore, the levels of IL-2-treated NK cell cytotoxicity from the lowest to highest in the co-cultures were as follows: NK(WT) + MO(WT) < NK(WT) + MO(KO) < NK(KO) + MO(WT) < NK(KO) + MO(KO) (Figure 3A).

Secretion of IFN-γ in the co-cultures of NK cells with monocytes followed the same trend as seen with cytotoxicity. NK cells from wild type mice cultured with wild type monocytes secreted the lowest amounts of IFN-γ when compared to those cultured with monocytes from *Cox-2^flox/flox^;LysM^Cre/^*^+^ mice, which secreted the next highest levels (Figure 3B). Significant amounts of IFN-γ were obtained when NK cells from *Cox-2^flox/flox^;LysM^Cre/^*^+^ mice were cultured either with wild type (*P* < 0.05) or *Cox-2^flox/flox^;LysM^Cre/^*^+^ monocytes (*P* < 0.05) (Figure 3B). IL-2-treated NK cells in the absence of monocytes from both wild type and *Cox-2^flox/flox^;LysM^Cre/^*^+^ mice did not secrete detectable IFN-γ (Figure 3B). Similarly, monocytes in the absence of NK cells did not secrete IFN-γ (Figure 3B).

### COX-2 gene deletion in mouse embryonic fibroblasts (MEFs) resulted in a significant susceptibility to NK cell-mediated lysis

Purified NK cells obtained from spleens of control WT littermates and *Cox-2^flox/flox^;LysM^Cre/^*^+^ mice were cultured with or without monocytes in the presence of IL-2 treatment. Afterward, the NK cells were used as effectors in a standard ^51^Cr release assay against wild type and COX-2^−/−^ MEFs from global COX-2^−/−^ mice. As shown in Figure 3C, NK cells from wild type mice cultured with and without autologous monocytes mediated lower levels of cytotoxicity against COX-2^−/−^ MEFs when compared to NK cells from *Cox-2^flox/flox^;LysM^Cre/^*^+^ mice cultured with and without autologous monocytes (*P* < 0.05), and lower cytotoxicity could be observed against wild type MEFs (*P* < 0.05). NK cells obtained from *Cox-2^flox/flox^;LysM^Cre/^*^+^ mice cultured with autologous monocytes had the greatest cytotoxicity against both wild type (*P* < 0.05) and COX-2^−/−^ MEFs (*P* < 0.05), although the highest levels were seen against COX-2^−/−^ MEFs when compared to wild type MEFs (*P* < 0.05) (Figure 3C).

### Co-culture with COX-2^−/−^ monocytes, but not COX-2^−/−^ T cells, increased the cytotoxic function of NK cells

NK cells and monocytes were purified from either control WT littermates or *Cox-2^flox/flox^;LysM^Cre/^*^+^ mice. T cells were purified from wild type or global COX-2 knockout mice. NK cells were treated with IL-2 and cultured alone or with purified CD3^+^ naïve T cells or monocytes from wild type or COX-2^−/−^ mice. Afterward, T cells and monocytes were removed from the co-cultures and NK cells were used as effector cells against wild type and COX-2^−/−^ MEFs in a standard ^51^Cr release assay (Figure 3C). The cytotoxic function of NK cells from wild type mice was lower against both wild type and COX-2^−/−^ MEFs, and the addition of either T cells or monocytes from wild type mice did not increase the cytotoxicity significantly (Figure 3C). NK cells obtained from *Cox-2^flox/flox^;LysM^Cre/^*^+^ mice cultured with autologous monocytes, but not with T cells from global COX-2^−/−^mice, increased cytotoxicity of NK cells significantly against both wild type (*P* < 0.05) and COX-2^−/−^ MEFs (*P* < 0.05). Albeit, the highest increase could be observed against COX-2^−/−^ MEFs when compared to wild type MEFs, while the addition of T cells from global COX-2 knockout mice did not have significant effect on NK cell cytotoxicity (Figure 3C).

### Dendritic cells derived from monocytes of *Cox-2^flox/flox^;LysM^Cre/+^* mice were more susceptible to NK cell-mediated cytotoxicity than dendritic cells from wild type mice

Dendritic cells were derived from purified monocytes by the addition of IL-4 and GM-CSF for 7 days. Differentiated DCs from wild type or *Cox-2^flox/flox^;LysM^Cre/^*^+^ mice were labeled with ^51^Cr and used as targets in a standard ^51^Cr release assay against IL-2-treated NK cells derived from wild type mice in the presence and absence of monocytes. As predicted, DCs differentiated from *Cox-2^flox/flox^;LysM^Cre/^*^+^ monocytes were more susceptible to IL-2-treated NK cell-mediated lysis as compared to those differentiated from monocytes obtained from control WT littermates (*P* < 0.05) (Figure 3D). NK cells purified from control WT mice cultured with monocytes from *Cox-2^flox/flox^;LysM^Cre/^*^+^ mice induced the highest lysis of *Cox-2^flox/flox^;LysM^Cre/^*^+^ DCs when compared to DCs from wild type mice (*P* < 0.05) (Figure 3D). Although NK cells obtained from wild type mice cultured with autologous monocytes lysed *Cox-2^flox/flox^;LysM^Cre/^*^+^ DCs more as compared to wild type DCs, the levels of cytotoxicity were significantly lower when compared to NK cells obtained from wild type mice and cultured with monocytes from *Cox-2^flox/flox^;LysM^Cre/^*^+^ mice (*P* < 0.05) (Figure 3D).

### LPS induced split anergy in murine NK cells as evident by a decrease in cytotoxicity and an increase in IFN-γ secretion by NK cells

**Figure 4:**
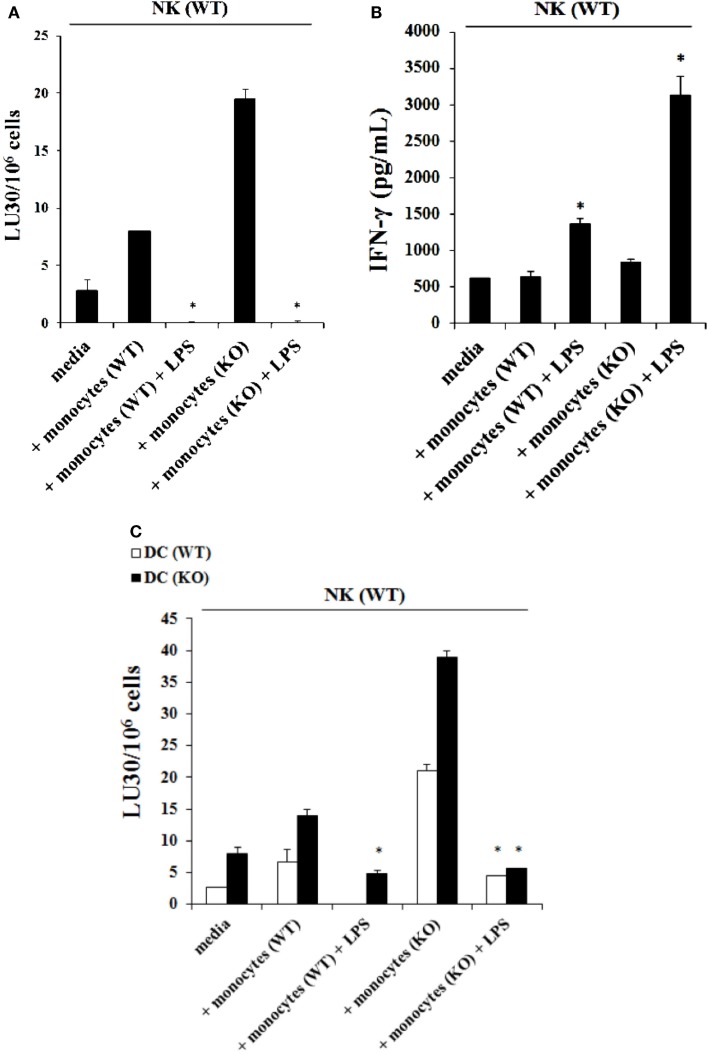
**The addition of LPS to NK cells cultured with monocytes induced split anergy in NK cells which resulted in significant inhibition of NK cell cytotoxicity but increased IFN-γ secretion**. IL-2-treated (1 × 10^4^ U/million) NK cells obtained from wild type mice were cultured with monocytes from wild type mice or *Cox-2^flox/flox^;LysM^Cre/^*^+^ mice for 7 days and then treated with or without LPS (20 ng/mL) for an additional day. Afterward, NK cells were used as effector cells in a standard 4 h ^51^Chromium release assay against YAC-1 cells. The lytic units 30/10^6^ cells were determined using inverse number of NK cells required to lyse 30% of the target cells × 100. *P < 0.05 was obtained for differences in cytotoxicity between untreated and LPS-treated NK cells cultured with monocytes from control littermates or those from *Cox-2^flox/flox;^LysM^Cre/^*^+^ mice **(A)**. NK cells were treated as described in (A) and afterward the supernatant was removed from the co-cultures and the levels of IFN-γ secretion were determined using specific ELISAs. *P < 0.05 was obtained for differences in secretion of IFN-γ between untreated and LPS-treated NK cells cultured with monocytes from control littermates or those from *Cox-2^flox/flox^;LysM^Cre/^*^+^ mice **(B)**. NK cells were prepared as described in (A) and used as effector cells against DCs derived from monocytes from either wild type or *Cox-2^flox/flox;^LysM^Cre/^*^+^ mice in a standard 4 h^51^Chromium release assay. The lytic units 30/10^6^ cells were determined using inverse number of NK cells required to lyse 30% of the target cells × 100 **(C)**. One of several representative experiments is shown in this figure.

Purified NK cells obtained from control WT littermates were treated with IL-2 and cultured in the presence of autologous monocytes or those obtained from *Cox-2^flox/flox^;LysM^Cre/^*^+^ mice in the absence and presence of LPS before they were used in a standard ^51^Cr release assay against YAC-1 tumors. The addition of LPS to the cultures of IL-2-treated NK cells with monocytes from control WT littermates resulted in a complete shutdown of the NK cells’ ability to lyse YAC-1 cells (*P* < 0.05) (Figure 4A) while it increased the amount of IFN-γ secreted by the NK cells (*P* < 0.05) (Figure 4B). Similarly, IL-2-treated NK cells obtained from control WT littermates cultured with monocytes from *Cox-2^flox/flox^;LysM^Cre/^*^+^ mice significantly increased NK cell-mediated cytotoxicity against YAC-1 cells when compared to control WT littermate NK cells cultured with autologous monocytes, and the addition of LPS completely abolished cytotoxicity (*P* < 0.05) (Figure 4A) while increasing IFN-γ secretion significantly (*P* < 0.05) (Figure 4B). Similar results to those seen with YAC-1 targets were also seen when control and *Cox-2^flox/flox^;LysM^Cre/^*^+^ DCs were used as targets (Figure 4C). As shown in Figure 4C, the addition of LPS to IL-2-treated NK cells from wild type mice either cultured with autologous monocytes or with monocytes obtained from *Cox-2^flox/flox^;LysM^Cre/^*^+^ mice resulted in decreased NK cell cytotoxicity against both wild type (*P* < 0.05) and *Cox-2^flox/flox^;LysM^Cre/^*^+^ DCs (*P* < 0.05), albeit the decrease was substantially more with *Cox-2^flox/flox^;LysM^Cre/^*^+^ DCs when compared to wild type DCs (Figure 4C). When NK cells from C57bl6 mice unrelated to the breeding colony control littermates for COX-2 were used in the presence and absence of autologous monocytes with and without LPS, similar results to those obtained with NK cells from control WT littermates in regards to cytotoxicity and IFN-γ secretion were seen (Figures S3A,B in Supplementary Material).

### Decreased constitutive expression of MHC class-I on COX-2^−/−^ MEFs and increased expression after treatment with IFN-γ and/or TNF-α

**Figure 5:**
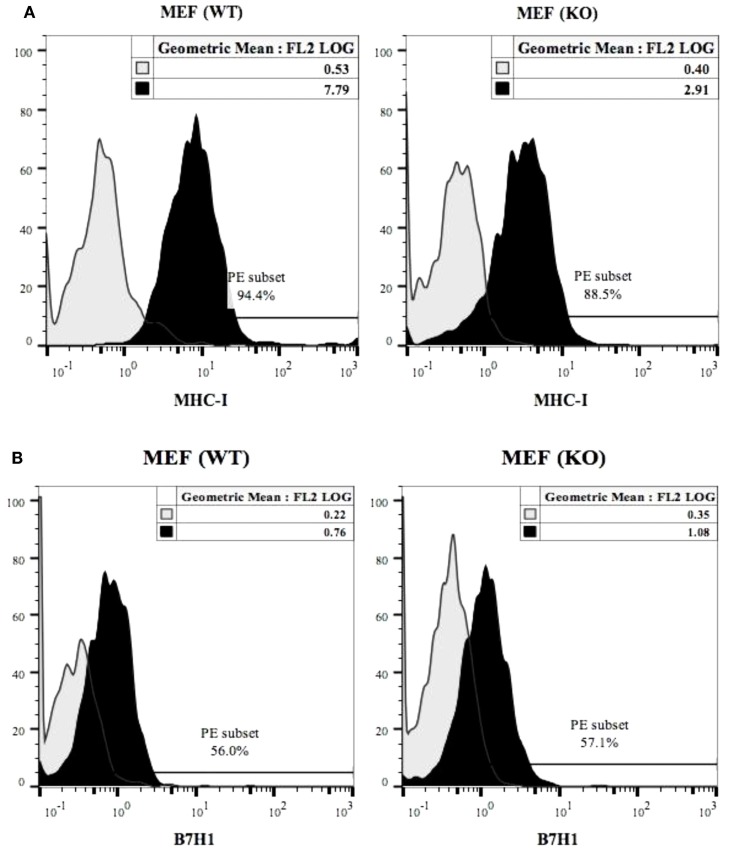

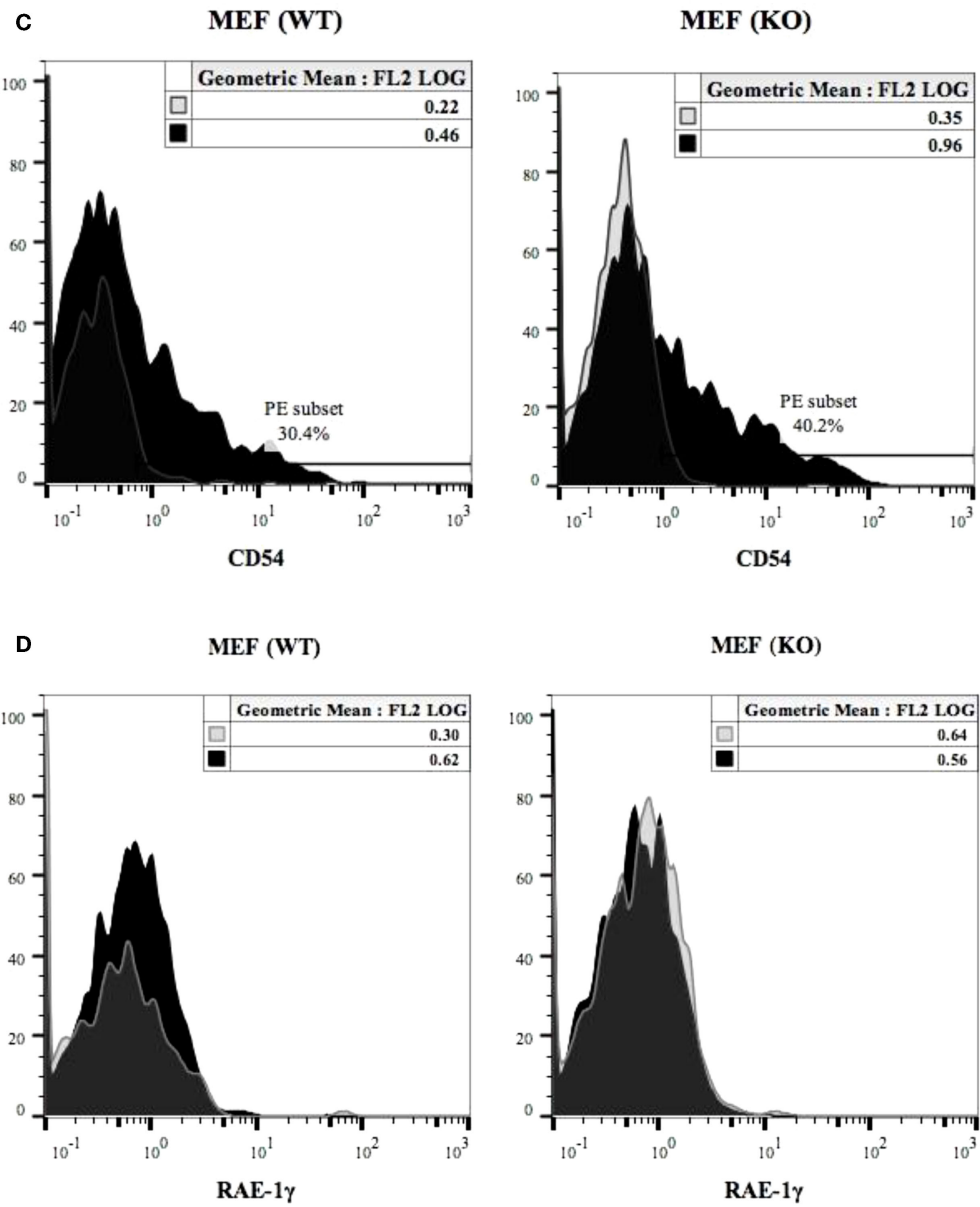
**MHC class-I, B7H1 and CD54 surface receptor analysis on wild type and COX-2 knockout MEFs**. The surface expression of MHC class-I **(A)**, B7H1 **(B)**, and CD54 **(C)** on wild type and COX-2 knockout MEFs were assessed using staining with PE-conjugated antibodies followed by flow cytometric analysis. The surface expression of Rae-1γ on wild type and COX-2 knockout MEFs was assessed using staining with PE-conjugated antibodies followed by flow cytometric analysis **(D)**. Isotype control antibody was used as control. One of four experiments is shown.

Expression of MHC class-I (Figure 5A), B7H1 (Figure 5B) and CD54 (Figure 5C) were determined on wild type and COX-2^−/−^ MEFs. COX-2^−/−^ MEFs demonstrated lower expression of MHC-class I but no significant change for B7H1 or CD54 expression for untreated MEFs (Figures 5A–C), whereas those treated with IFN-γ expressed higher MHC class-I, B7H1 and CD54 surface receptors when compared to wild type MEFs (Figures S4A–C in Supplementary Material). The addition of TNF-α to COX-2^−/−^ MEFs increased MHC class-I and CD54 but had no effect on B7H1 expression when compared to wild type MEFs (Figures S4A–C in Supplementary Material). Treatment with the combination of IFN-γ and TNF-α synergistically increased MHC class-I, B7H1, and CD54 on both wild type and COX-2^−/−^ MEFs, however, the levels of expression were higher on COX-2^−/−^ MEFs when compared to wild type MEFs (Figures S4A–C in Supplementary Material). Decrease in constitutive expression of MHC class-I on untreated COX-2^−/−^ MEFs were seen for the majority of the experiments, however, its modulation with IFN-γ and/or TNF-α were variable, demonstrating an increase on COX-2^−/−^ MEFs as compared to wild type MEFs in most experiments (Figures S4A–C in Supplementary Material); but in a few experiments a decrease rather than an increase was noted on COX-2^−/−^ MEFs as compared to wild type MEFs which depended on cell passage number and growth dynamics (data not shown). No expression of Rae-1γ could be seen on either wild type or COX-2−/− MEFs (Figure 5D).

### Significant down-modulation of NK cell receptors after their culture with MEFs and monocytes

**Figure 6:**
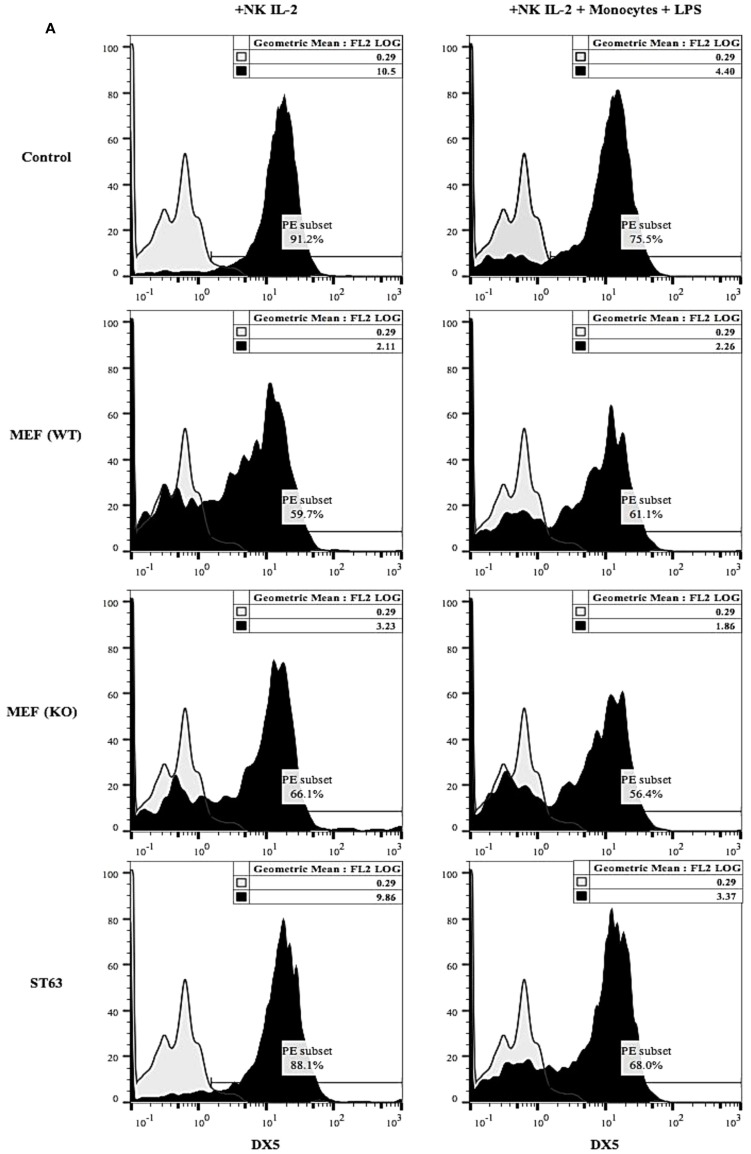

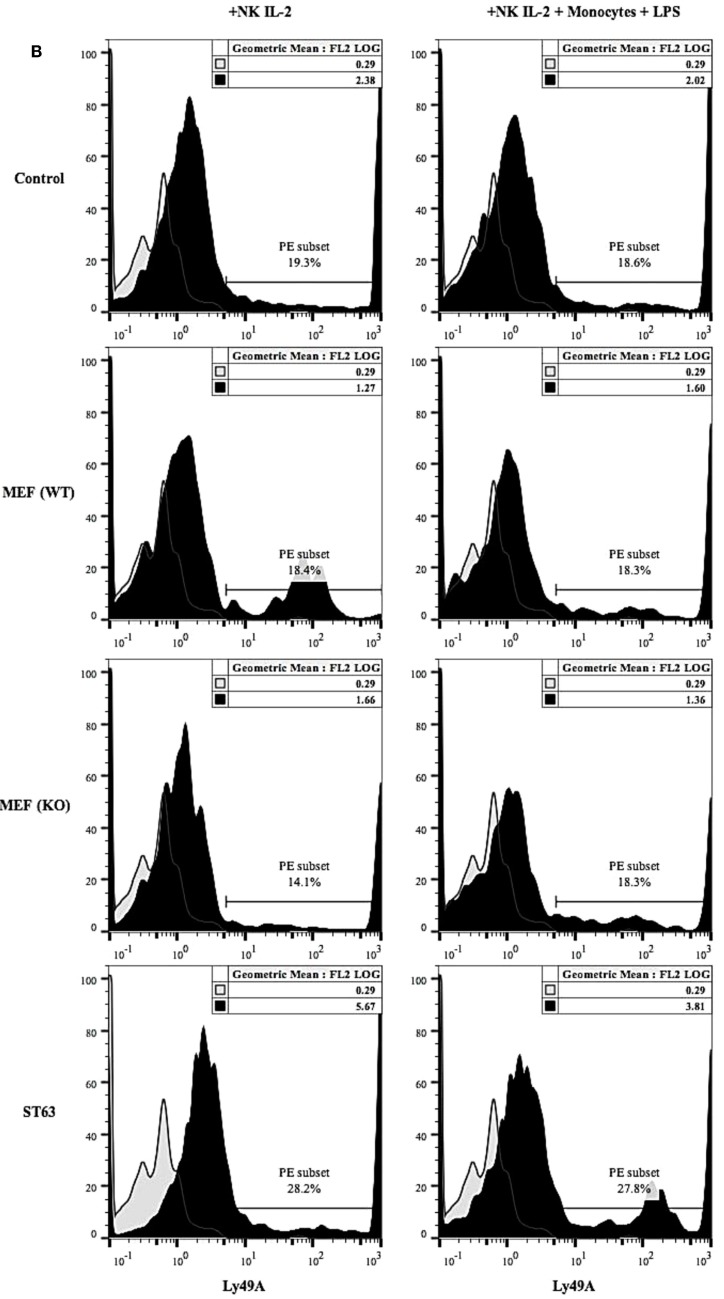

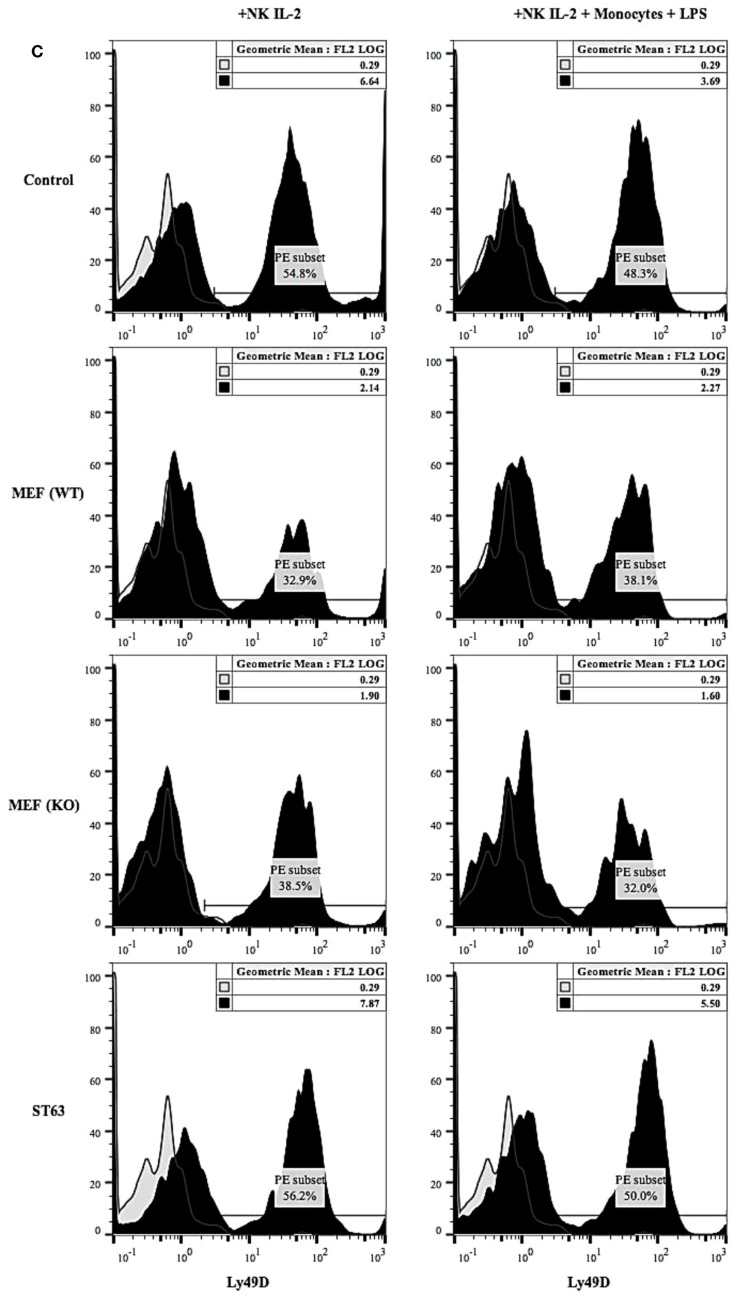

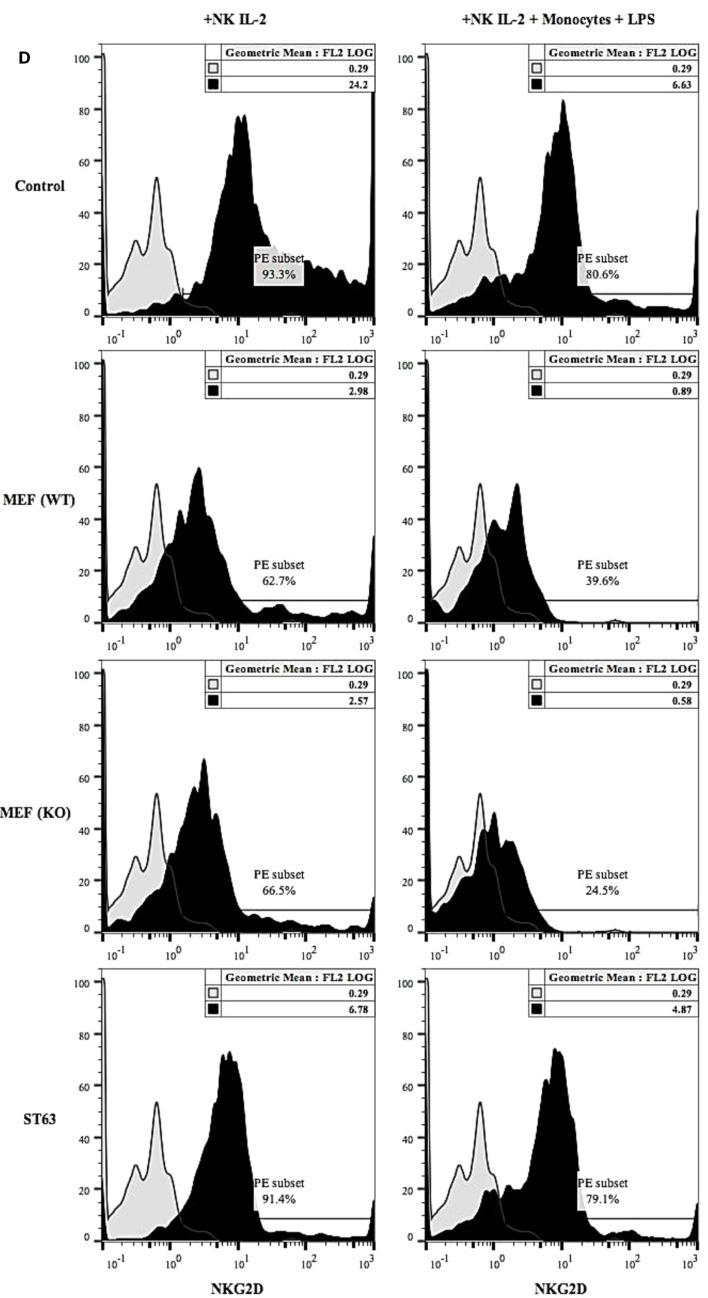
**Receptor analysis of purified splenic NK cells co-cultured with MEFs from wild type and COX-2 knockout mice**. Purified NK cells were treated with IL-2 (10,000 U/mL) and cultured without and with monocytes (NK: monocytes, 2:1) and LPS (100 ng/mL) for 24 h. Afterward, NK cells were co-cultured with either wild type or COX-2 knockout MEFs or ST63 at 9:1 (NK cells: target ratio) for 48 h. Thereafter, the surface expression of DX5 **(A)**, Ly49A **(B)**, Ly49D **(C)** and NKG2D **(D)** were assessed using staining with PE-conjugated antibodies followed by flow cytometric analysis. Isotype control antibody was used as control. One of three experiments is shown.

The expression of DX5 (Figure 6A), Ly49A (Figure 6B), Ly49D (Figure 6C), and NKG2D (Figure 6D) were determined on the surface of NK cells activated with IL-2 and cultured with and without monocytes and LPS in the presence and absence of wild type and COX-2^−/−^ MEFs or ST63. A generalized decrease in all four receptor expression on NK cells were noted after culture with wild type or COX-2^−/−^ MEFs (Figure 6), whereas the expression of DX5 and NKG2D was either decreased or not changed on NK cells after interaction with ST63. In contrast, an increase in the expression of Ly49A and Ly49D was seen on NK cells cultured with ST63 cells (Figure 6). Culture of NK cells with monocytes also exhibited significant down-modulation of all four receptors in the absence and presence of culture with wild type and COX-2^−/−^ MEFs and ST63 cells (Figure 6).

### Monocytes in the presence and absence of LPS induced split anergy in IL-2 treated human NK cells

**Figure 7:**
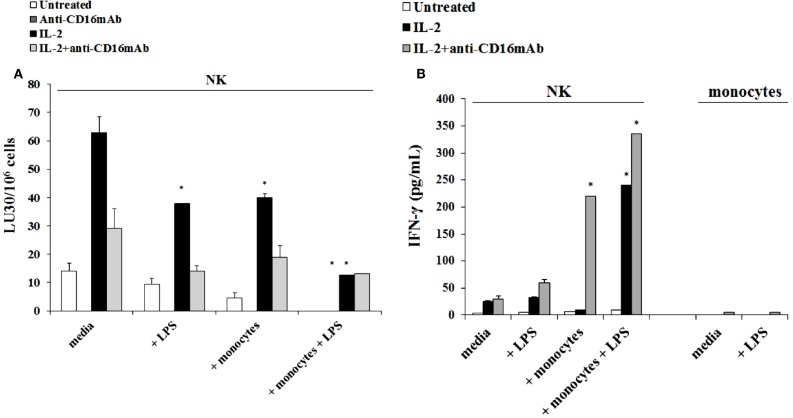
**Induction of split anergy mediated by LPS was observed in Human NK cells which resulted in a loss of their cytotoxic function but gained the ability to secrete high levels of IFN-γ, especially in the presence of autologous monocytes**. Human NK cells were purified from healthy donors and were left untreated or treated with IL-2 (1000 U/mL), anti-CD16mAb (3 μg/mL), or the combination of IL-2 (1000 U/mL) and anti-CD16mAb (3 μg/mL) in the presence or absence of LPS (20 ng/mL) and autologous monocytes (NK cell:monocytes, 1:1) for 24–48 h. Afterward, the cytotoxicity against OSCSCs was assessed using a standard 4 h ^51^Chromium release assay. Percent cytotoxicity was obtained at different effector to target ratio and the lytic units 30/10^6^ cells were determined using inverse number of NK cells required to lyse 30% of the tumor cells × 100 **(A)**. NK cells were prepared as described in Figure 5A. Monocytes were treated with IL-2 (1000 U/mL) and/or anti-CD16mAb (3 μg/mL) and LPS (20 ng/mL) for 24–48 h and used as controls. After the treatment period, the supernatants were removed from the co-cultures and the levels of IFN-γ cytokine were measured with specific ELISA **(B)**. *P < 0.05 was obtained for the differences in cytotoxicity and IFN-γ secretion between NK cells cultured in media and those treated with LPS, monocytes, or the combination of LPS and monocytes. One of several representative experiments is shown in this figure.

**Figure 8:**
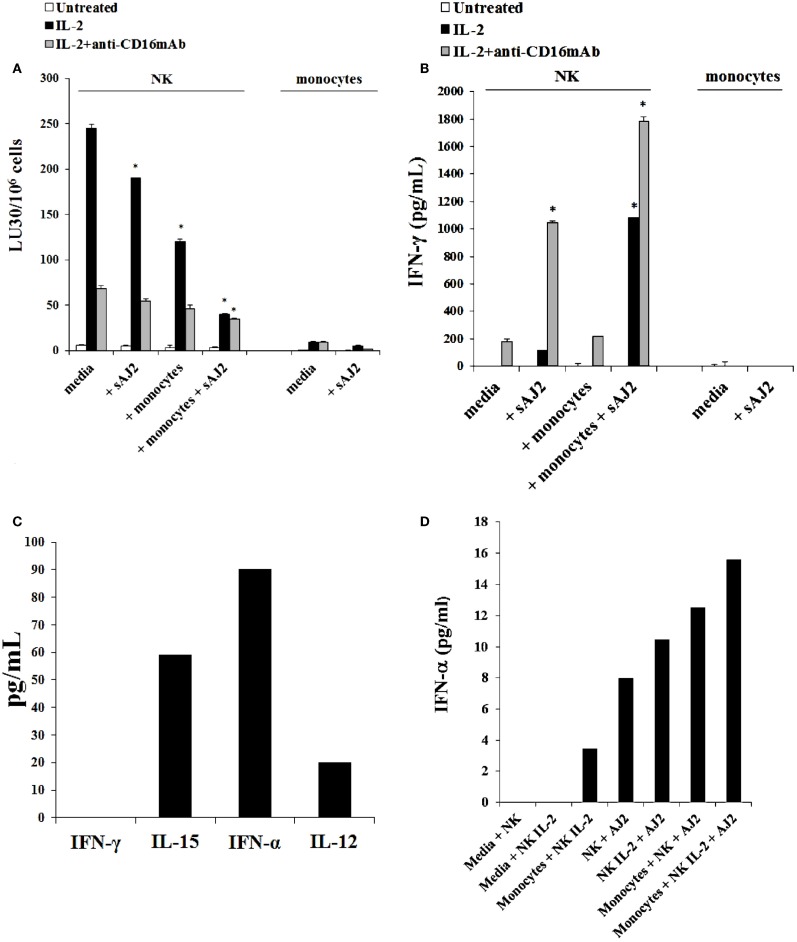
**Split anergy induced by sAJ2 and monocytes also occurred in Human NK cells**. Human NK cells were purified from healthy donors and left untreated or treated with IL-2 (1000 U/mL) or the combination of IL-2 (1000 U/mL) and anti-CD16mAb (3 μg/mL) in the presence of sAJ2 (NK cell:sAJ2, 1:3), autologous monocytes (NK cell:monocytes, 1:1) or the combination of sAJ2 (NK cell:sAJ2, 1:3) and autologous monocytes (NK cell:monocytes, 1:1) for 24–48 h. Afterward, the cytotoxicity against OSCSCs cells was assessed using a standard 4 h ^51^Chromium release assay. Percent cytotoxicity was obtained at different effector to target ratio and the lytic units 30/10^6^ cells were determined using inverse number of NK cells required to lyse 30% of the tumor cells X100 **(A)**. NK cells were prepared as described in Figure 6A and after the treatment period, the supernatants were removed from the co-cultures and the levels of IFN-γ cytokine were measured with specific ELISA. *P < 0.05 was obtained for the differences in cytotoxicity and IFN-γ secretion between human NK cells cultured in media and those treated with sAJ2, monocytes or the combination of sAJ2 and monocytes. One of several representative experiments is shown in this figure **(B)**. Purified NK cells were cultured with autologous monocytes (NK cell:monocytes, 1:1). After an overnight incubation, the supernatants were collected and the levels of IFN-γ, IL-15, IFN-α, and IL-12 were determined by ELISAs in a multiplexed format using Luminex technology **(C)**. Untreated and IL-2 stimulated NK cells were treated with monocytes and sAJ2 as described in (A). Afterward, the supernatants were collected and the level of IFN-α was determined by ELISAs in a multiplexed format using Luminex technology **(D)**.

The addition of LPS, as well as sAJ2, to human NK cells in the absence and presence of monocytes resulted in the significant induction of split anergy (Figures 7 and 8). As demonstrated in Figure 7, LPS induced loss of NK cell-mediated cytotoxicity against Oral Squamous Carcinoma Stem Cells (OSCSCs) while increasing IFN-γ secretion. Unlike mouse NK cells in which culture of monocytes with IL-2-treated NK cells increased NK cell cytotoxicity and secretion of IFN-γ, culture of monocytes with IL-2-treated NK cells from humans inhibited cytotoxicity (*P* < 0.05) while increasing IFN-γ secretion (*P* < 0.05) (Figure 7). The highest decrease in cytotoxicity and increase in IFN-γ secretion were observed when IL-2 or IL-2 and anti-CD16mAb-stimulated NK cells cultured with monocytes were treated with LPS (*P* < 0.05) (Figure 7). Split anergy in human NK cells was also induced by gram-positive bacteria sAJ2 (Figure 8). The loss of cytotoxicity in IL-2-treated NK cells was induced with the addition of monocytes in the presence or absence of sAJ2 while it induced significant secretion of IFN-γ (*P* < 0.05) (Figures 8A,B). The highest decrease in cytotoxicity and increase in IFN-γ secretion was obtained when IL-2 or IL-2 and anti-CD16mAb-treated NK cells were cultured with monocytes and treated with sAJ2 (*P* < 0.05) (Figures 8A,B). In addition to IFN-γ, the levels of IL-6, IL-8, IL-10, GM-CSF, and TNF-α were also increased when NK cells were cultured with monocytes and bacteria (Figure S5 in Supplementary Material). No release of MICA or MICB could be seen in the cultures of NK cells with monocytes (data not shown), even though the same treatment induces significant IL-6 and IL-8 release in the co-cultures of NK cells with monocytes (Figure S6 in Supplementary Material). Therefore, although monocytes increased IFN-γ secretion in both species, they inhibited cytotoxicity by human NK cells whereas they increased cytotoxicity by mouse IL-2-treated NK cells. Treatment of NK cells and monocytes with LPS, on the other hand, inhibited cytotoxicity in both human and mouse NK cells while increasing IFN-γ secretion substantially. Human monocytes secreted significant levels of NK activating cytokines IL-15, IFN-α, and IL-12 (Figure 8C) and the levels of IFN-α increased when cultured with the NK cells (Figure 8D).

## Discussion

Table 1 provides a short list of genes which upon deletion in cells trigger inflammation and augment immune cell function in mice and in *in vitro* culture models. Specifically, the deletion of NF-κB in tumors was found to increase NK cell-mediated cytotoxicity and secretion of IFN-γ significantly (2, 3), and induce auto-immunity and inflammation *in vivo* (63, 64). Moreover, conditional knockout of STAT3 in hematopoietic cells was found to result in the induction of colitis in mice due to chronic gut inflammation (57, 65). Furthermore, we have shown previously that NK cells target poorly differentiated cells or stem cells with lower expression of many important differentiation receptors (45, 60, 66). In our recent studies, we reported that the stage of maturation and differentiation of healthy untransformed stem cells, as well as transformed tumorigenic cancer stem cells, is predictive of their sensitivity to NK cell-mediated lysis. In this regard, we have shown that stem-like/poorly differentiated oral and pancreatic tumors are significantly more susceptible to NK cell-mediated cytotoxicity; whereas, their differentiated counterparts are significantly more resistant (45). Based on these results, we have proposed, and recently demonstrated, that NK cells play a significant role in differentiation of the cells by providing critical signals via secreted cytokines as well as direct cell–cell contact (60). In addition, we have previously shown that human monocytes induce significant split anergy in NK cells (55, 57, 61, 65, 66). Induction of split anergy in NK cell effector function is thought to ultimately aid in driving differentiation of healthy, as well as transformed stem cells (55, 57, 61, 65, 66). Therefore, from these studies, and those listed in Table 1, it appears that inhibition of key molecules that take part in differentiation, or induction of de-differentiation in the cells are likely means of activating immune cells, particularly NK cells, in order to drive both selection and differentiation of their interacting cells. Since COX-2 is elevated during differentiation in many cells, we aimed at determining whether blocking COX-2 in monocytes or MEFs will also able to activate NK cell function.

The findings from our laboratory indicated that NK cells may sense the absence of key receptors on stem cells in order to aid in stem cell differentiation. To mediate this process, we hypothesized that NK cells will have to first receive signals which will allow them to undergo split anergy resulting in a decrease in their cytotoxic function and an increase in the production of cytokines primarily, IFN-γ and TNF-α, to promote differentiation of the cells (60). Increasing the ability of NK cells to not only be great effectors of selection of stem cells, but also great inducers of their differentiation has a significant physiological role in maintenance of homeostasis during health (6). The *in vivo* relevance of such observations was recently obtained in our laboratory (manuscript in prep).

The availability of targeted COX-2 knockout in myeloid compartment in mice provided the means to assess the function of NK cells in *ex vivo* culture assays with knockout of COX-2 in monocytes. To determine whether blocking COX-2 in monocytes allows NK cells to become activated and to study the consequences of such activation, we tested the activity of autologous NK cells after interaction with wild type and COX-2 knockout monocytes. In addition, several differences in the mode and consequence of activation between mouse and human NK cells were found. Targeted knockout of COX-2 in murine myeloid cells increased both the cytotoxicity and cytokine secretion capabilities of NK cells after their interaction with monocytes and DCs. Monocytes from control littermates were also able to activate NK cell functions but at a much lower level. Similarly, knockout of COX-2 in MEFs was able to increase both NK cell functions when compared to wild type MEFs.

The addition of LPS-mediated significant split anergy in NK cells by inhibiting cytotoxicity while increasing IFN-γ secretion after interaction either with monocytes or DCs. Thus, in mice split anergy in NK cells occurred after treatment with LPS and not with monocytes. In contrast, human monocytes, irrespective of whether they were activated with LPS or not, mediated significant split anergy in NK cells and the levels further increased when treated with LPS. Therefore, LPS induces split anergy in both murine and human NK cells with or without monocytes; however, only human monocytes induce split anergy in NK cells in the absence of LPS. The differences observed in induction of split anergy between mice and human NK cells after interaction with monocytes could be due to prior *in vivo* priming of NK cells in humans and not in mice. Indeed, human NK cells can kill susceptible targets even without prior activation with IL-2 and they require only a short period of stimulation with IL-2 to become highly activated to efficiently lyse many tumors. In contrast, murine NK cells do not mediate cytotoxicity without prior activation, and they require a longer period of activation with IL-2 to mediate cytotoxicity against susceptible targets. It is only when there is an activating event, such as that seen during interaction with knockout COX-2 cells that increased murine NK cytotoxicity can be seen after IL-2 activation. Therefore, in the case of human NK cells, monocytes may induce split anergy in *in vivo* primed NK cells, whereas monocytes from mice may provide the priming signals for naïve NK cells. However, both murine and human NK cells will undergo significant split anergy when treated with LPS.

In accordance with our findings, LPS was shown to induce IFN-γ production in purified IL-2 activated NK cells in the presence of a decrease in NK cell degranulation (67). However, the mechanism of NK cell activation by LPS is unclear at present. Kanevskiy et al. was unable to detect significant surface expression of TLR4 on NK cells, whereas all CD56^dim^ NK cells were found to be TLR4-positive in previous studies (67, 68). Thus, it was speculated that LPS may interact with receptors other than TLR4 or with intracellular TLR4 in NK cells.

Knockout of COX-2 in both monocytes and MEFs activate the function of NK cells significantly, whereas knockout of COX-2 in T cells was unable to activate NK cell functions. At present it is not clear why COX-2 knockout T cells are not able to activate NK cells. Moreover, the mechanisms by which COX-2^−/−^ monocytes and MEFs are able to increase NK cell function are not known yet. Interestingly, constitutive MHC class-I expression is lower on COX-2^−/−^ MEFs when compared to wild type MEFs, however, treatment with IFN-γ and/or TNF-α increases expression of MHC class-I, B7H1, and CD54 on COX-2^−/−^ MEFs more than wild type MEFs. Thus, it is possible that lower MHC class-I expression on COX-2^−/−^ MEFs (30–63% decrease) may partly be responsible for the increased activation of NK cells, however, whether such decrease in MHC class-I expression observed on COX-2^−/−^ MEFs is adequate to significantly contribute to activation of NK cell function is not clear at present. In addition, the dynamics of MHC class-I modulation on COX-2^−/−^ MEFs may be different during interaction with NK cells, since cytokines induced by NK cells may elevate the expression of MHC class-I on COX-2^−/−^ MEFs more and result in the faster cessation of NK cell activation when compared to wild type MEFs. Therefore, it is possible that mechanisms other than or in combination with that mediated by decreased MHC class-I are responsible for the activation of NK cells by COX-2^−/−^ MEFs.

It is likely that NK activating cytokines induced by monocytes such as IFN-α, IL-12, IL-15, and IL-18 (Figure 8C and data not shown), which are increased during their interaction with NK cells (Figure 8D) synergistically contribute to the increased activation of NK cells by wild type and COX-2^−/−^ monocytes. Moreover, we have also recently observed that monocytes upon activation substantially decrease expression of MHC class-I (manuscript submitted). Therefore, lack of inhibitory signals received from MHC class-I, compounded by increased cytokine signaling could be the mechanisms contributing to the increased activation of NK cells during interaction with monocytes. However, it is likely that activating potential of MEFs may be limited when compared to monocytes due to their lack of secretion of NK activating cytokines indicated above. Whether COX-2^−/−^ monocytes in contrast to wild type monocytes increase more of the NK activating cytokines during their interaction with NK cells, and thus contribute to increased activation of NK cells requires further investigation.

When murine NK cells were cultured with wild type and COX-2^−/−^ MEFs significant decreases in NKG2D, DX5, Ly49A, and Ly49D were observed, and no significant differences could be ascertained between those cultured with wild type or COX-2^−/−^ MEFs. In contrast, increased Ly49A and Ly49D expression on NK cells after interaction with ST63 could be observed, even though a decrease or no change could be seen for the expression of NKG2D and DX5, respectively. Interestingly, monocytes cultured with NK cells also mediated significant decrease in most NK cell receptors. Whether such decrease in NK cell receptors is due to ligand mediated binding and/or active inhibition by MEFs and monocytes regardless of ligand binding requires further investigation. Indeed, we were unable to observe any increase in the expression of Rae-1γ, one of the ligands for NKG2D on MEFs. Whether down-modulation of Ly49A inhibitory receptor plays a role in activating the function of NK cells, or triggering of activating Ly49D or NKG2D via ligands other than Rae-1γ play significant role in activating NK cell function during their interaction with COX-2^−/−^ MEFs or monocytes should await future investigation.

Both gram-positive and gram-negative strains of bacteria are capable of inducing significant split anergy in NK cells during co-culture with monocytes. Split anergized NK cells are key mediators of cell differentiation since they secrete significant levels of IFN-γ and TNF-α, which we have previously shown to drive differentiation of healthy, as well as transformed stem cells, in the absence of NK cell-mediated cytotoxicity (60). In addition to IFN-γ and TNF-α, a number of key pro-inflammatory and anti-inflammatory cytokines are highly induced during interaction of NK cells and monocytes with bacteria which could provide additional stimuli for potent activation of NK cells (Figure S5 in Supplementary Material).

Once stem cells are differentiated by the NK cells they are no longer targeted by the NK cells. Indeed, differentiation of OSCSCs and DPSCs by the NK cells is found to not only induce resistance to NK cell-mediated cytotoxicity, but also inhibit cytokine and chemokine secretion by the NK cells resulting in inhibition of inflammation (60). Promotion of differentiation and resolution of inflammation by NK cells may provide important mechanisms for the prevention of auto-immunity and chronic inflammation. In this regard, our recent results suggest that probiotic bacteria induced NK cell-mediated differentiation is important for the prevention of inflammation and maintenance of gut homeostasis (manuscript in preparation).

Our results collectively indicate that any disturbance in genes which are important for differentiation of the cells may be the cause of activation of NK cells and the maintenance of NK cells in an activated state. If such genes are deleted in monocytes which are potent activators of NK cells, they may maintain NK cells in an activated state, resulting in increased inflammation, and induction of inflammation induced tumors.

## Author Contributions

HCT contributed to design, data acquisition, analysis, interpretation of the data and preparation of the manuscript. AA, AK, KK and PT contributed to design, data acquisition, analysis and interpretation of the data. AJ contributed to conception, design, analysis, interpretation of the data and preparation of the manuscript.

## Conflict of Interest Statement

The authors declare that the research was conducted in the absence of any commercial or financial relationships that could be construed as a potential conflict of interest.

## Supplementary Material

The Supplementary Material for this article can be found online at http://journal.frontiersin.org/article/10.3389/fimmu.2015.00259/abstract
