# Supplementary material for: Differential Cytotoxicity but Augmented IFN-γ Secretion by NK Cells after Interaction with Monocytes from Humans, and Those from Wild Type and Myeloid-Specific COX-2 Knockout Mice
Source: Front Immunol. 2015 Jun 9;6:259. doi: 10.3389/fimmu.2015.00259 (PMC4460808; doi:10.3389/fimmu.2015.00259)

**Supplementary figures**

**Supplemental Figure 1.** **Flow cytometric analysis of splenic NK cells and Bone marrow derived monocytes obtained from wild type animals**

A representative flow cytometric analysis of purified NK cells from spleen and purified monocytes from bone marrow of wild type mice, as described in Materials and Methods, is shown in this figure. Percentages of NK cells and monocytes before and after purification were determined using staining with FITC-CD45 and PE-DX5 antibodies for NK cells and FITC-CD45 and PE-F4/80 antibodies for monocytes followed by flow cytometric analysis. Isotype control antibodies were used as controls. The numbers in the quadrants 2 and 4 are the percentages for DX5 or F4/80 positive cells within CD45 subset.

**Fig. S1**

**
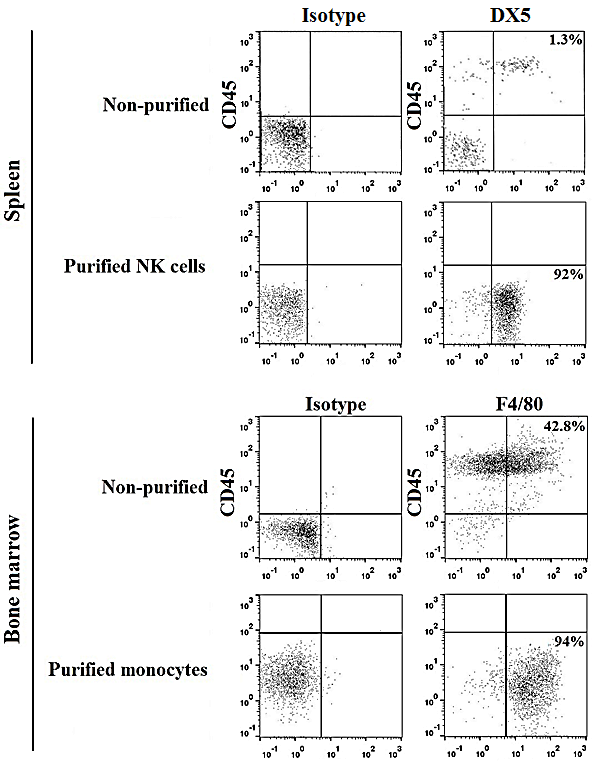
**

**Supplemental figure 2.** **Increased cytotoxicity by NK cells derived from *Cox-2^flox/flox^;LysM^Cre/+^* mice as compared to control littermates**

Purified NK cells obtained from either control (WT) or *Cox-2^flox/flox^;LysM^Cre/+^* (KO) mice were left untreated or treated with IL-2 (1X10^4^ units/million) for 7 days before the cells were used against YAC-1 cells (**A**) and ST63 (**B**) cells in a standard 4 hour ^51^Chromium release assay. Different effectors to target ratios were used to assess cytotoxicity.

**
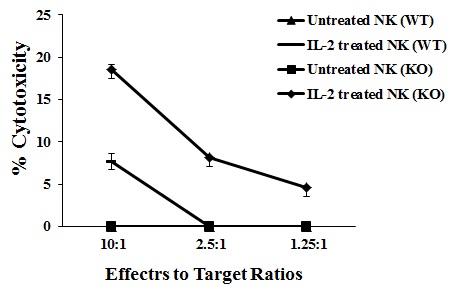
Fig. S2A**

**
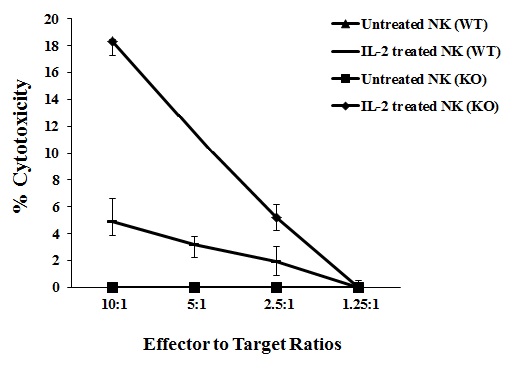
Fig. S2B**

**Supplemental Figure 3. Monocytes and LPS induced split anergy in NK cells derived from C57BL/6 wild type mice**

NK cells purified from C57BL/6 wild type mice were treated with IL-2 (1X10^4^ units/million) with or without LPS (1μg/mL) in the presence or absence of autologous monocytes (NK cells: monocytes, 5:1) for 5 days. Afterwards, NK cells were used as effector cells in a standard 4 hour ^51^Chromium release assay against ST63 cells. The lytic units 30/10^6^ cells were determined using inverse number of NK cells required to lyse 30% of the target cells X100 **(A)**. NK cells were prepared as described in Fig. S3A. Autologous purified monocytes were left untreated or treated with IL-2 (1X10^4^ units/million) or the combination of IL-2 (1X10^4^ units/million) and LPS (1μg/mL) and used as control. Afterwards, the supernatants were removed from the co-cultures and the levels of IFN-γ secretion were determined using specific ELISAs **(B)**. * The differences between IL-2 treated NK cells and those treated with monocytes or the combination of LPS and monocytes in cytotoxicity and secretion of IFN-γ are significant at P<0.05. One of several representative experiments is shown in this figure.

**Fig. S3A Fig. S3B**


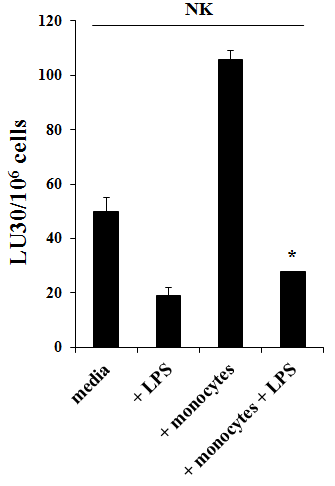

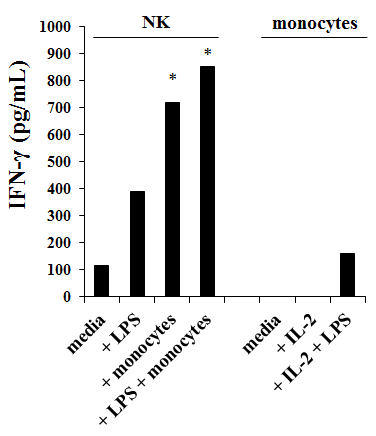


**Supplemental Figure 4. MHC class I, B7H1 and CD54 surface analysis of wild type and COX-2 knockout MEFs**

Wild type (WT) and COX-2 knockout (KO) MEFs were left untreated or treated with IFN-γ (10ng/mL), TNF-α (10ng/mL) or their combination for 24 hours. Afterwards, the surface expression of MHC class-1 **(A)**, B7H1 **(B)**, and CD54 **(C)** (dark histograms) were assessed using staining with PE-conjugated antibodies followed by flow cytometric analysis. Isotype control antibodies (light histograms) were used as controls. One of four experiments is shown.

**
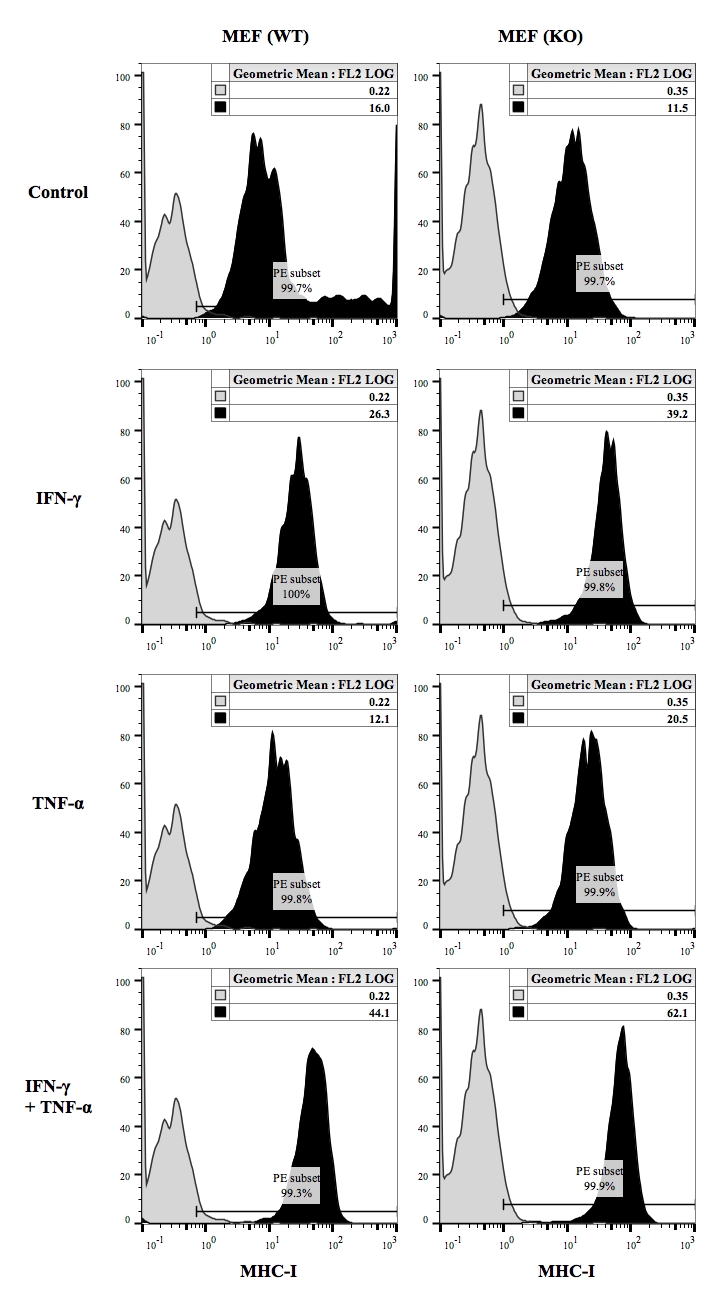
Fig. S4A**

**
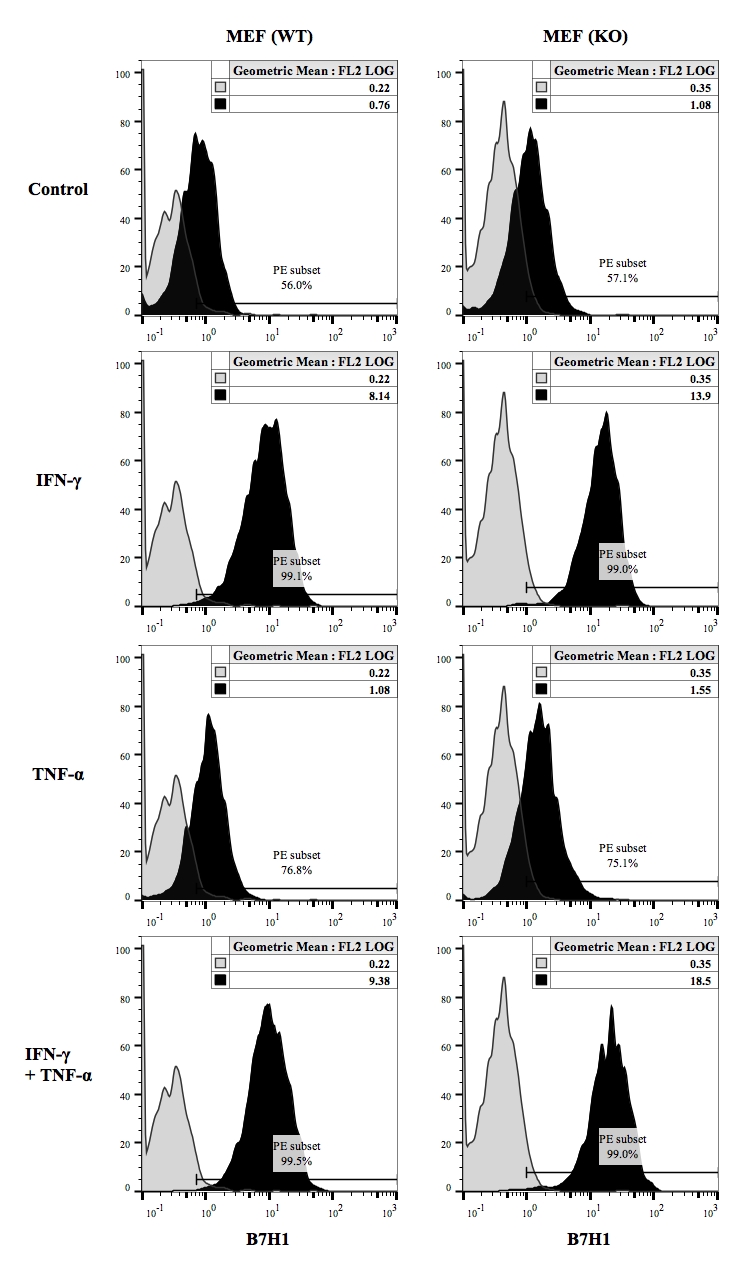
Fig. S4B**

**
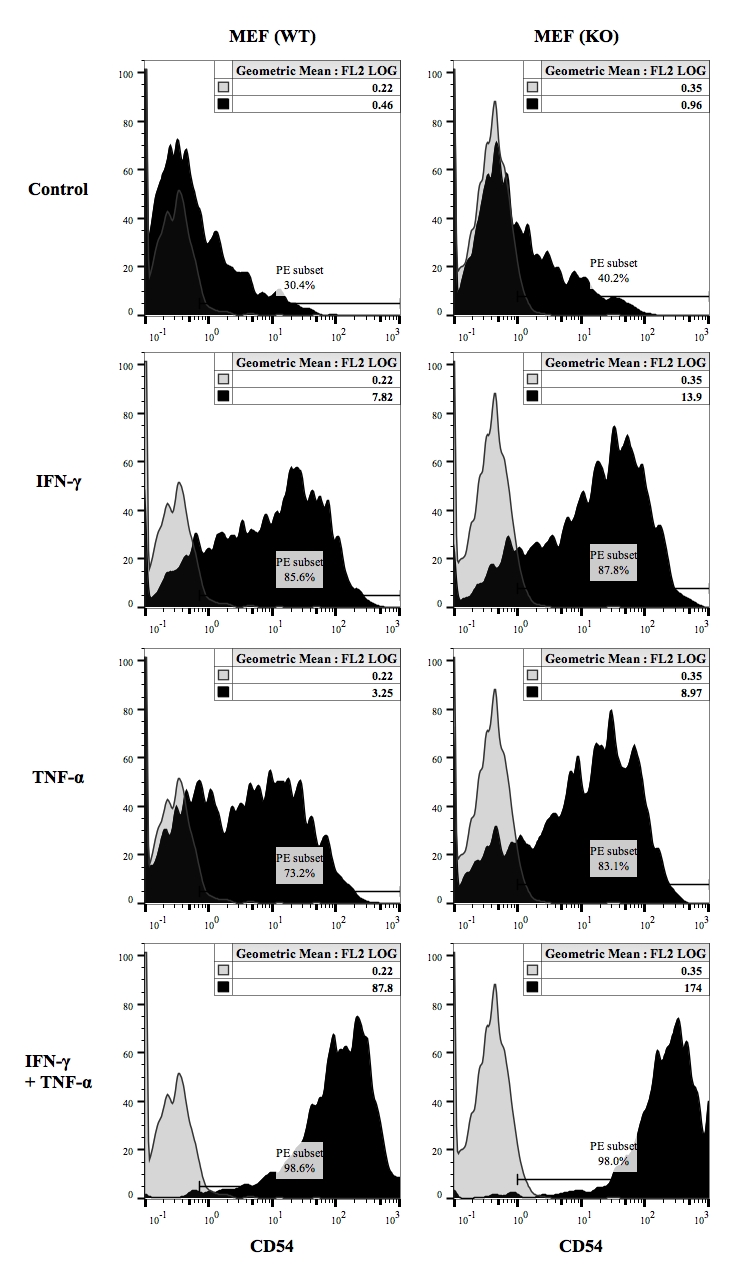
Fig. S4C**

**Supplemental Figure 5.** **Human monocytes triggered significant secretion of pro-inflammatory and anti-inflammatory cytokines in co-cultures with NK cells and probiotic bacteria**


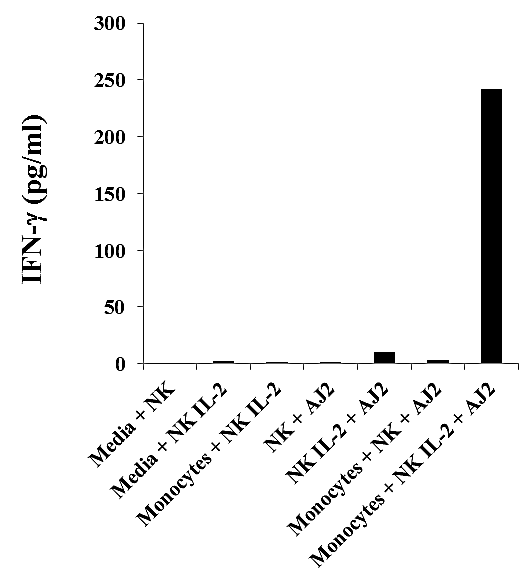

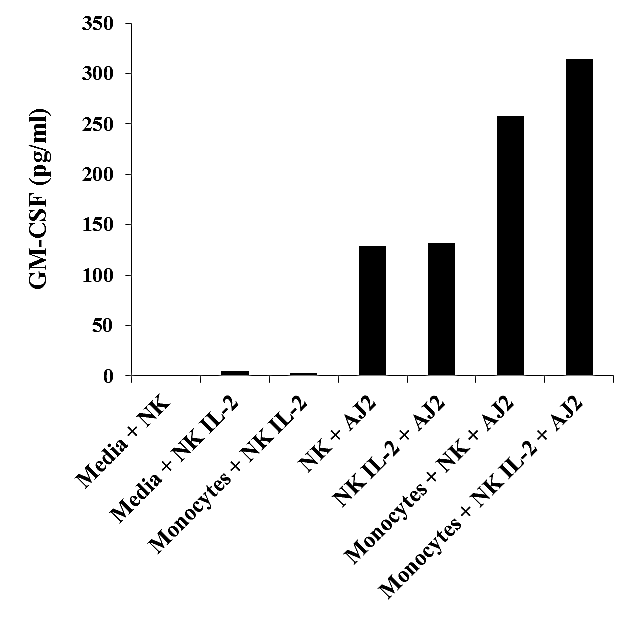
NK cells were left untreated or treated with IL-2 (1000 units/mL) in the presence or absence of sAJ2 bacteria with or without autologous monocytes (NK cell:monocytes:bacteria 1:1:2) for 48 hours, after which supernatants were removed and the released cytokines were determined using multiplex Luminex analysis.


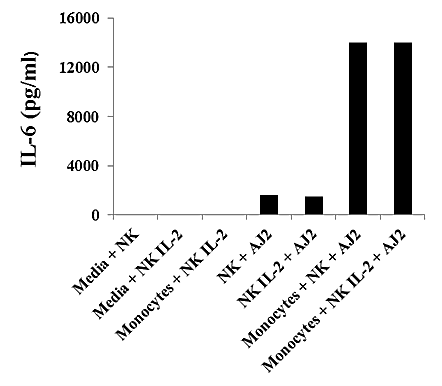

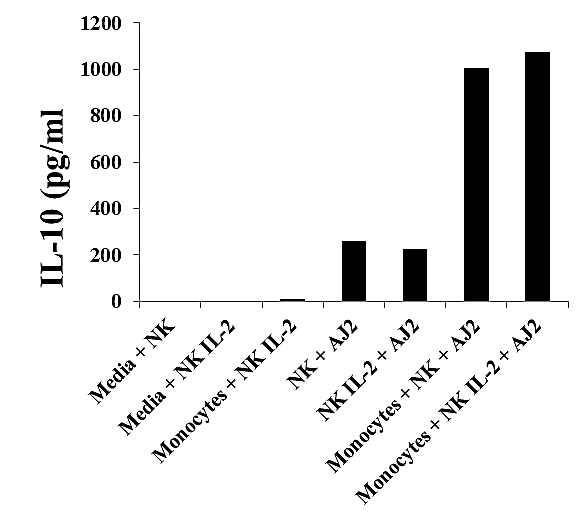


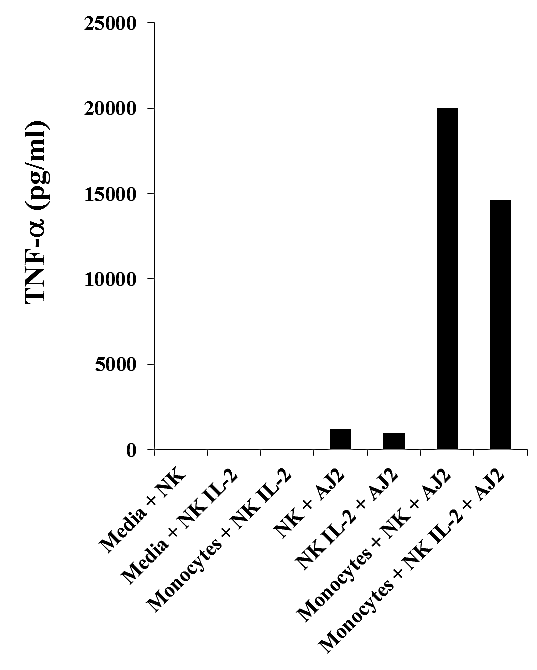


**Supplemental figure 6.** **Human monocytes potentiated the secretion of IL-6 and IL-8 in co-cultures with NK cells**

NK cells were left untreated or treated with IL-2 (1000 units/mL), anti-CD16mAb (3μg/mL) or the combination of IL-2 (1000 units/ml) and anti-CD16mAb (3μg/ml) in the presence or absence of autologous monocytes (NK cell:monocytes, 1:1) for 48 hours, after which supernatants were removed and the released cytokines and chemokines were determined using multiplex Luminex analysis.


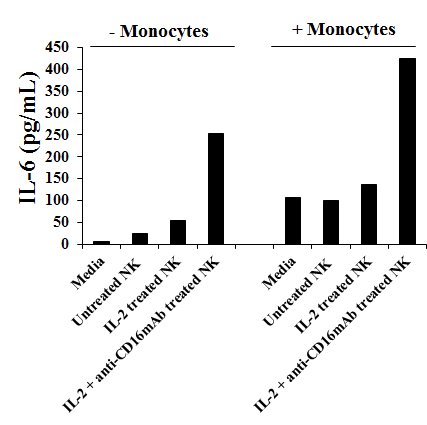

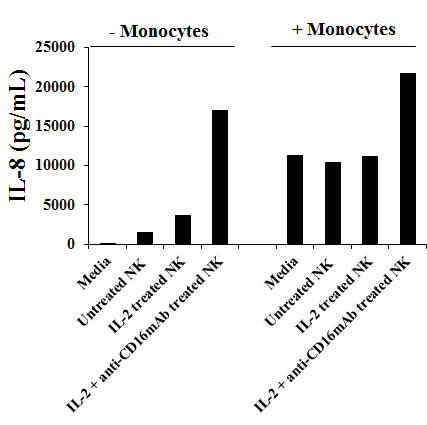

Supplement: Supplementary file 1 [file Data_Sheet_1.DOCX]
